# The validity of transdiagnostic factors in predicting homotypic and heterotypic continuity of psychopathology symptoms over time

**DOI:** 10.3389/fpsyt.2023.1096572

**Published:** 2023-05-19

**Authors:** Bori Jung, Hyunsik Kim

**Affiliations:** Department of Psychology, Sogang University, Seoul, Republic of Korea

**Keywords:** transdiagnostic factors, continuity, internalizing–externalizing, comorbidity, predictive validity

## Abstract

Studies of the continuity of psychopathology symptoms mainly involved the traditional conceptualization that mental disorders are discrete entities. However, high comorbidity rates suggest a few transdiagnostic factors that underlie individual disorders. Therefore, the present study examined the validity of transdiagnostic factors in predicting homotypic and heterotypic continuity of comorbidity classes across two waves in a nationally representative sample. We conducted a latent transition analysis to investigate how transdiagnostic factors differentially affect the transition probabilities of comorbidity classes across time. Results found a notable predictive validity of transdiagnostic factors: (a) internalizing strongly predicted the stability of the internalizing class and transition from the externalizing class to internalizing class, and (b) externalizing predicted the transition from the internalizing class to externalizing class. The study also found a more dynamic prediction pattern leading to equifinality and multifinality of psychopathology symptoms. The findings suggest that transdiagnostic factors can provide information on how individuals’ symptom manifestations change over time, highlighting the potential benefits of incorporating transdiagnostic factors into assessment, treatment, and prevention.

## 1. Introduction

Studies examining the course of psychopathology have indicated that mental disorder symptoms change over a person’s lifetime (1–4), with two broad patterns in the stability and change of mental disorder symptoms: homotypic and heterotypic continuity. Homotypic continuity refers to the stability of symptoms of a given disorder over time (e.g., current depressive symptoms predicting subsequent depression). On the other hand, heterotypic continuity refers to the phenomenon in which symptoms of individuals develop into different patterns of behaviors at a later time (e.g., current depressive symptoms transitioning to subsequent substance use disorders).

Previous studies on the continuity of psychopathology used a categorical approach (e.g., DSM5), conceptualizing mental disorders as dichotomous and distinct entities (5–9). This approach considers homotypic continuity as the stability of a particular disorder diagnosis (i.e., diagnostic stability) over time (e.g., the stability of panic disorder). In contrast, heterotypic continuity is referred to as the transition from a given disorder to another disorder (e.g., the transition from panic disorder to a major depressive disorder). However, the categorical approach’s major caveat is the high comorbidity rates (10, 11). Given the high comorbidity rates, individuals whose patterns of psychopathology symptoms follow “pure” homotypic continuity (e.g., an individual presenting only a panic disorder diagnosis and continuously experiencing panic disorder only) would be very rare. Moreover, heterotypic continuity attributed to the change of comorbidity patterns over time may obscure the examination of the transition from one disorder to another. This limitation implies a need for a better understanding of an alternative approach to the mechanisms of homotypic and heterotypic continuity of psychopathology.

Given the high frequency of comorbidities among mental disorders (10–14), a transdiagnostic approach to psychopathology has emerged to overcome the shortcomings of the categorical method (15–17). The transdiagnostic approach poses several higher-order psychopathology dimensions (i.e., transdiagnostic factors) to describe the source of the common variance among various mental disorders. This approach assumes that latent transdiagnostic factors underlie phenotypic symptoms of various psychopathologies. Given this, the covariation and commonalities among those symptoms/diagnoses are accounted for by these latent transdiagnostic factors, and particular mental disorder diagnoses and/or phenotypic symptoms are considered partial manifestations of high-order transdiagnostic factors. Numerous transdiagnostic factors have been identified and hierarchically organized into the framework of the Hierarchical Taxonomy of Psychopathology (HiTOP) model (18). This model organizes psychopathologies from narrow to broad dimensions, where higher levels of factors reflect the overall degree of commonalities, while lower factors reflect the distinctiveness of symptoms/diagnoses encompassed by the model (19, 20). This hierarchical arrangement allows researcher to (a) understand the association between phenotypic variation and its correlates (e.g., risk factors and clinical outcomes) by addressing psychopathology dimensionally (21), and (b) investigate psychopathology at multiple levels with varying specificity (22, 23).

Early studies of the transdiagnostic approach proposed a two-factor internalizing–externalizing (INT–EXT) model (15, 17, 24) to delineate the comorbidity structure of common mental disorders. That is, high correlations and comorbidities among common mental disorder diagnoses can be accounted for by the presence of two higher-order factors, internalizing and externalizing: the internalizing factor represents a broad tendency to experience symptoms of internalizing psychopathology and explains the shared features among mood and anxiety disorders. [e.g., major depressive disorder (MDD), panic disorder, and generalized anxiety disorder (GAD)]. The externalizing factor represents the commonalities among externalizing-type disorders [e.g., substance abuse disorder (SUD), conduct disorder (CD), antisocial personality disorder (ASPD)] and encompasses disinhibited and antagonistic features (25). Subsequent studies proposed a bifurcated INT–EXT model where the internalizing factor further splits into distress and fear subfactors (16, 26–28). However, studies comparing the unitary and bifurcated INT–EXT models documented mixed results (29, 30). Given that numerous prior studies have replicated the two-factor INT–EXT model across various samples and populations (26, 31–39), the current study explored homotypic and heterotypic continuity of psychopathology symptoms in the context of the INT–EXT model; that is, we focused on the stability of and transition between internalizing and externalizing disorders over time.

Prior studies investigating the relationships between transdiagnostic factors and the continuity of psychopathology revealed that internalizing and externalizing factors predicted the stability of psychopathology symptoms over time (40, 41). Specifically, Lahey et al. (42) described the effect of the commonalities among mental disorders on their continuity. The researchers hypothesized that if cross-sectional correlations between different mental disorders (i.e., time1X–time1Y) were significantly associated with the longitudinal correlations between them (i.e., time1X–time2Y), it would indicate that the common variance among mental disorders was affecting the comorbidity at one time point and the longitudinal continuity of the mental disorders. Indeed, there was a significant positive correlation (ρ = 0.86) between the magnitude of cross-sectional associations among mental disorders and their longitudinal associations (42). This finding indicates that mental disorders tend to vary relative to the extent to which they correlate (i.e., the commonalities among related disorders; the transdiagnostic factors). These findings imply that transdiagnostic factors might account for the pattern of homotypic and heterotypic continuity of mental disorder symptoms over time.

The transdiagnostic approach indicates that (a) each particular mental disorder is a partial manifestation of a higher-order factor and (b) a given transdiagnostic factor plays a vital role in the continuity of the disorders that load on the factor. Given this, we re-conceptualized homotypic continuity as the stability of and/or transition between particular disorders within a given transdiagnostic domain. For instance, while the categorical approach considers a transition from MDD to GAD as heterotypic continuity, the transdiagnostic approach considers such a phenomenon as homotypic continuity within an internalizing dimension, assuming that both disorders are temporal manifestations of the underlying core factor of internalizing. In addition, the internalizing factor accounts for the “transition” between the two disorders within the internalizing domain. Hence, we redefine heterotypic continuity as the transition of one disorder to another across INT–EXT dimension (e.g., transition from an internalizing-type disorder to an externalizing-type disorder and vice versa).

Therefore, the current study investigated how transdiagnostic factors predicted the stability of and transition between different comorbidity classes over time, based on the conceptualization that transdiagnostic factors represent one’s liability to experiencing different forms of mental disorders within a given dimension (e.g., the internalizing factor denotes one’s vulnerability to internalizing psychopathologies, such as mood and anxiety disorders). While variable-centered approaches are gaining traction, there have been few investigations examining person-centered approaches to mental disorder comorbidity and the transdiagnostic factors’ validity in predicting patterns of homotypic and/or heterotypic continuity. Thus, firstly, we used latent class analysis (LCA) to investigate whether individuals could be classified into distinct comorbidity classes that aligned with the results obtained from the dimensional approaches. That is, we were interested in exploring whether a person-centered approach to conceptualizing and modeling psychopathology and comorbidity would provide converging or diverging evidence compared to the dimensional approach. Although prior studies have suggested different class models of comorbidity [e.g., three-class (43, 44), four-class (45), five-class (46), and seven-class models (47)], we focused on a three-class model (i.e., internalizing, externalizing, and low psychopathology) since prior studies that employed a three-class model identified individuals whose psychopathology symptom profiles were characterized by particularly increased levels of internalizing and/or externalizing disorders. Secondly, we examined whether transdiagnostic internalizing and externalizing factors predicted patterns of homotypic (i.e., stability of an internalizing or externalizing class over time) or heterotypic continuity (i.e., a transition from an internalizing class to an externalizing class, or vice versa) using the latent transition analysis (LTA) framework.

General findings on predictive value of transdiagnostic factors have revealed that transdiagnostic internalizing and externalizing are predictive of similar problems later on (48–50). For instance, transdiagnostic internalizing and externalizing factors accounted for the majority of positive associations between primary and secondary comorbid disorders, with time-lagged associations being stronger for within-domain disorders than between-domain (50). Additionally, baseline total internalizing problems measured by the Child Behavior Checklist (CBCL) selectively predicted a wide range of mood and anxiety disorders at a 5-year follow-up (48). A study by Mesman and Koot (49) also found that early preschool internalizing and externalizing problems were predictors of DSM-IV counterpart diagnoses 8 years later, even after controlling for the effect of family risk factors. Altogether, these findings seem to support that earlier internalizing and externalizing problems are predictors for later psychopathology. Given the predictive value of transdiagnostic factors for subsequent mental disorder symptoms, it is possible that an individual’s transdiagnostic factor levels account for the continuity of a given disorder and/or transition between different disorders over time (29, 51). For instance, individuals with high internalizing factor levels would be likely to experience a manifestation of later internalizing symptoms (e.g., a transition from EXT to INT) or prolongation of current internalizing symptoms (e.g., INT to INT) over time. Conversely, individuals with high externalizing factor levels would be likely to experience a manifestation of subsequent externalizing symptoms (e.g., a transition from INT to EXT) or prolongation of current externalizing problems (e.g., EXT to EXT) across time.

**FIGURE 1:**
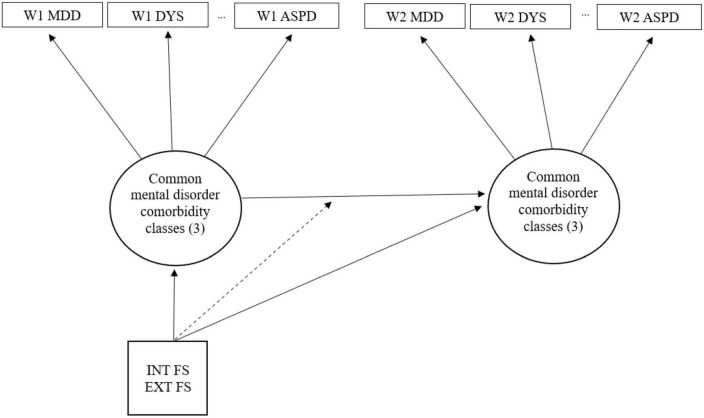
Latent transition model where INT FS and EXT FS moderate the transition between common mental disorder comorbidity classes. INT FS, internalizing factor scores; EXT FS, externalizing factor scores.

In sum, our study aimed to investigate the validity of transdiagnostic factors in predicting homotypic and/or heterotypic continuity of psychopathology symptoms over time using a nationally representative sample of the United States (i.e., the National Epidemiologic Survey on Alcohol and Related Conditions) (Figure 1). Lahey et al. (42) investigated the continuity of psychopathology using the same dataset and found that patterns of continuity across time reflect correlations between different mental disorders (i.e., the transdiagnostic structure). Kim and Eaton (40, 52) conducted additional studies on the same sample and found the structural stability of transdiagnostic constructs, both in variable-centered and person-centered analyses. However, to our knowledge, no study has addressed the fundamental question of how internalizing and externalizing factors predict homotypic and/or heterotypic continuity of different comorbidity classes over time. We hypothesized that transdiagnostic factors would predict the homotypic and heterotypic continuity of internalizing and externalizing classes across time differently. Specifically, we expected that internalizing would predict the stability of an internalizing class and the transition from an externalizing class to an internalizing class over time. As such, we anticipated that externalizing would predict the continuity of an externalizing class and change from an internalizing class to an externalizing class over time.

## 2. Materials and methods

### 2.1. Participants

We analyzed the data from 34,653 participants—non-institutionalized U.S. adults aged 18–90 years—who responded to both waves of the National Epidemiologic Survey on Alcohol and Related Conditions (NESARC). The dataset included two longitudinal waves conducted between 2001–2002 (Wave 1) and 2004–2005 (Wave 2). The response rate of Wave 1 was 81.0%, Wave 2 was 86.7%, and the cumulative response rate was 70.2%. The sample consisted of 58% women, with self-identified race as follows: White (58.2%), African American (19.0%), Hispanic or Latino (18.3%), Asian/Native Hawaiian/Pacific Islander (2.8%), and American Indian/Alaska Native (1.7%). A total of 58.5% of the sample attained some college education or higher, 27.5% had a high school education, and 14.0% had less than high school education. The data were weighted to represent the population from the 2000 census, adjusting for age, gender, and race/ethnic. Informed consents were obtained from all NESARC respondents prior to participation. The research protocol received approval from the United States Census Bureau and the Office of Management and Budget.

### 2.2. Assessment

Experienced lay interviewers assessed past-year and lifetime DSM-IV psychiatric disorders with the Alcohol Use Disorder and Associated Disabilities Interview Schedule [AUDADIS-IV (53, 54)]. Past-year psychiatric disorders were assessed based on symptoms within 12 months from each wave (Wave 1 and Wave 2), and lifetime psychiatric disorders were measured only once based on all the symptoms experienced before Wave 1. Psychiatric disorders included three mood disorders (MDD, dysthymic disorder, and bipolar I disorder), four anxiety disorders (GAD, panic disorder, social phobia, and specific phobia), four substance use disorders (alcohol, nicotine, marijuana, and other drug dependence), and ASPD. We categorized relatively rare drug dependence disorders, including opioids, amphetamines, hallucinogens, inhalants, heroin, sedatives, tranquilizers, cocaine, and solvents, into one variable (i.e., “other drug dependence”) for a sufficient statistical variance for analyses. ASPD was only assessed once at Wave 1. However, given that personality disorders are stable over time according to the DSM, and we need externalizing disorder indicators other than substance use disorder (SUD) for modeling the transdiagnostic externalizing factor, we also included the diagnosis of ASPD as one of the externalizing dimensions’ indicators. The reliabilities of the AUDADIS-IV mood and anxiety disorders and ASPD diagnoses were fair to good, with kappa ranging from 0.40–0.67, and reliabilities of SUDs were good, with kappa ranging between 0.54 and 0.76 (53–56). The test–retest reliabilities for the AUDADIS-IV alcohol abuse and dependence, nicotine dependence, MDD, and dysthymia were good, with kappa ranging between 0.58 and 0.74, and were fair to good for anxiety disorders, with kappa ranging between 0.40 and 0.52 (57). Internal consistency of the other drug dependence variable was good in a previous study [α = 0.77 (37)].

### 2.3. Statistical analysis

#### 2.3.1. Modeling transdiagnostic internalizing and externalizing factors

We used lifetime diagnoses as indicators to measure one’s liability to internalizing and externalizing disorders (i.e., transdiagnostic internalizing and externalizing factors) based on the following prior literature. First, according to the transdiagnostic approach to psychopathology, each of particular mental disorders are conceptualized as temporal manifestations of one’s broad vulnerability to psychopathology (18, 19, 58) and the stability and continuity of mental disorder symptoms can be attributed to one’s levels of transdiagnostic factors (40, 41, 59). Similarly, the developmental progression hypothesis argues that (a) individuals’ psychopathology will progress differently over time depending on one’s levels of liability to psychopathology and (b) individuals with elevated liability, when followed long enough, are likely to move in and out of different diagnostic categories over time (60, 61). Given these, it is probable that the commonalities among related *lifetime* diagnoses reflect one’s broad vulnerability to internalizing and externalizing psychopathologies (36), which likely predicts the stability of mental disorder symptoms over time. These studies support our conceptualization that lifetime transdiagnostic factors represent liability factors.

Given that researchers have replicated the unitary internalizing–externalizing model across various samples (26, 31, 33, 37, 39) supported by numerous studies (15, 24, 27, 62), the framework guided the building of internalizing and externalizing factors in this study. When modeling internalizing and externalizing factors, we initially considered the following three models: (a) a confirmatory factor analysis (CFA) model allowing a correlation between internalizing and externalizing factors, (b) CFA model constraining internalizing and externalizing factors to be uncorrelated, and (c) exploratory structural equation modeling [ESEM (63)] model with an orthogonal rotation extracting internalizing and externalizing factors in a data-driven manner (Supplementary Table 1 reports the fit indices for each model). We eventually chose the ESEM model based on prior studies that reported the superiority of ESEM relative to CFA in model fit and factor differentiation (63–65); using ESEM can result in improved model fit and less correlated factors, increasing the discriminant validity of factors. Moreover, the uncorrelated CFA model showed a poor data fit. However, despite its good data fit, we did not choose the correlated CFA model because we were interested in investigating the independent contribution of internalizing and externalizing in predicting the continuity of psychopathology while controlling for the overlap between the two. In sum, we estimated individuals’ internalizing and externalizing factor levels using ESEM with orthogonal Crawford–Ferguson family varimax rotation [CF-V (66)].

Since modeling internalizing and externalizing factors directly in the LTA framework yielded non-convergence issues, we extracted internalizing and externalizing factor scores and saved them using ESEM. Then, we used the saved factor scores in LTA (see section “Latent transition analysis” for details). Next, we treated lifetime diagnoses as categorical with weighted least squares with a mean and variance adjustment (WLSMV) estimator. Finally, we evaluated model fit using root mean square error of approximation (RMSEA), a comparative fit index (CFI), and the Tucker-Lewis Index (TLI) with the value of CFI and TLI ≥ 0.95 and RMSEA ≤ 0.08 indicating good model fit (67). Estimated internalizing and externalizing factor scores showed individuals’ standing along the continuous higher-order dimensions. The scores also represented one’s liability for internalizing or externalizing disorders.

#### 2.3.2. Latent class analysis

Latent class analysis (68) identifies different subgroups of individuals that share certain characteristics, a person-centered approach focusing on the relationships between groups of individuals. We conducted LCA to identify homogenous subgroups of individuals with distinct comorbidity patterns. While, in general, exploratory LCA involves sequentially extracting an increasing number of classes and identifying an optimal class based on fit indices, our aim was not to identify an optimal class solution. Instead, we chose to conduct a three-class model because (a) we were interested in identifying internalizing and externalizing classes at each wave to investigate the stability of and transition between those classes over time and (b) prior studies of mental disorder comorbidity reported that a three-class LCA solution yielded internalizing, externalizing, and low psychopathology classes (43, 44). We used past-year diagnoses assessed at each wave as indicators of comorbidity classes. We conducted LCA using a robust maximum likelihood estimator [MLR (69)], treating past-year diagnoses as categorical.

#### 2.3.3. Latent transition analysis

Latent transition analysis is a longitudinal extension of LCA which estimates transition probabilities between latent classes over time. LTA involves two models: (1) measurement and (2) structural. The measurement model encompasses cross-sectional LCAs at each wave to identify subgroups of individuals with distinct patterns (e.g., comorbidity classes). Then, the structural model estimates the transition probabilities of latent classes over time. In LTA, transition probabilities vary as a function of covariates that moderate the relationship between latent classes (70), and the impact of covariates is estimated using multinomial logistic regression. We anticipated that one’s transdiagnostic factor levels would affect the transition probabilities of latent classes over time. For example, an individual’s likelihood of transitioning from a class at Wave 1 to another class at Wave 2 would vary depending on the individual’s internalizing and/or externalizing factor levels (i.e., covariates). Thus, the LTA structural model estimates the transition probabilities influenced by one’s transdiagnostic internalizing and externalizing levels.

In interpreting the result of LTA, homotypic continuity is referred to as the stability of internalizing or externalizing classes (e.g., Wave 1 INT to Wave 2 INT) over time, and heterotypic continuity is referred to as the transition from an internalizing class to an externalizing class and vice versa. We conducted LTA using past-year diagnoses assessed at each wave after controlling for age and gender. In addition, we included the transdiagnostic factor scores (i.e., internalizing and externalizing) estimated and saved through ESEM as a predictor for transition probabilities between different latent comorbidity classes across waves (71). We used Mplus version 7.4 (69) for all analyses.

## 3. Results

### 3.1. Internalizing and externalizing factor scores

The two-factor INT–EXT model estimated through ESEM exhibited a good data fit (CFI = 0.983, TLI = 0.974, RMSEA = 0.018). All mood and anxiety disorders loaded on internalizing from 0.50 to 0.83, and ASPD and all SUDs loaded on externalizing from 0.64 to 0.83. The factor loadings for the INT–EXT model are in Table 1.

**TABLE 1:** Factor loadings for exploratory structural equation modeling of INT–EXT.

Lifetime diagnosis	MDD	DYS	BIP	PAN	SOPH	SPEPH	GAD	ASPD	ALCOD	NICD	MARD	OTHDD
INT FS	0.83	0.80	0.59	0.59	0.60	0.50	0.77	0.25	0.15	0.21	0.26	0.28
EXT FS	0.25	0.22	0.42	0.27	0.23	0.24	0.18	0.64	0.76	0.65	0.80	0.80

MDD, major depressive disorder; DYS, dysthymic disorder; BIP, bipolar disorder I; PAN, panic disorder; SOPH, social phobia; SPEPH, specific phobia; GAD, generalized anxiety disorder; ASPD, antisocial personality disorder; ALCOD, alcohol dependence; NICD, nicotine dependence; MARD, marijuana dependence; OTHDD, other drug dependence; INT FS, internalizing factor scores; EXT FS, externalizing factor scores. We used Crawford–Ferguson family varimax rotation to conduct exploratory structural equation modeling with weighted least squares means and variance-adjusted estimation.

### 3.2. Latent classes of common mental disorder comorbidity: a three-class model

**FIGURE 2:**
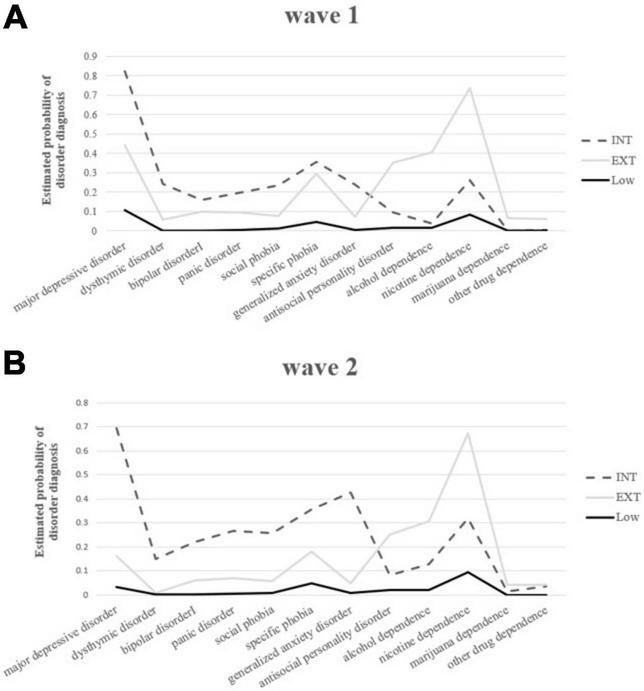
**(A)** Profiles of parameter estimates for latent classes derived from the three-solution latent class analysis (LCA) model of common mental disorders at Wave 1. **(B)** Profiles of parameter estimates for latent classes derived from three-solution LCA model of common mental disorders at Wave 2. INT, internalizing class; EXT, externalizing class; Low, low psychopathology class.

The three-class LCA model at each wave identified highly similar comorbidity classes over time (Figure 2). These included (a) a low psychopathology class characterized by low probabilities of all psychiatric disorders, (b) an internalizing class characterized by particularly elevated mood and anxiety disorders, and (c) an externalizing class characterized by elevated ASPD and all SUDs.

### 3.3. Transitions between classes across waves

The estimated transition probabilities between latent classes are in Table 2. Individuals in the low psychopathology class demonstrated extremely high probabilities (94.7%) of staying in the same class from Wave 1 to Wave 2. Those in the externalizing class showed relatively low but still high probabilities (84.7%) of remaining in the same class across waves (i.e., homotypic continuity). Individuals in the internalizing class showed the lowest probabilities (29.3%) of staying in the same class over time. When the individuals from the internalizing class transitioned to other comorbidity classes, they were most likely to transition to the low psychopathology class (66.5%), and only a small subset (4.2%) transitioned to the externalizing class (i.e., heterotypic continuity). Subjects in the externalizing class showed relatively low probabilities of transitioning to other comorbidity classes, with a probability of 11.1% of transition to the low psychopathology class and 4.2% to the internalizing class (i.e., heterotypic continuity).

**TABLE 2:** Estimated transition probabilities in three-solution LTA of common mental disorder comorbidity across two waves.

	Wave 2		
	INT	EXT	Low
Wave 1			
INT	0.293	0.042	0.665
EXT	0.042	0.847	0.111
Low	0.034	0.019	0.947

We conducted a three-solution latent transition analysis (LTA) with past-year psychiatric disorders at both waves, including internalizing factor scores, externalizing factor scores, age, and gender as covariates. Row headings represent three classes at Wave 1. Column headings represent three classes at Wave 2. INT, internalizing class; EXT, externalizing class; Low, low psychopathology class.

### 3.4. The validity of transdiagnostic factors in predicting homotypic and heterotypic continuity

Table 3 summarizes the results of the multinomial logistic regression analysis. We focused on and interpreted the predictive validity of the transdiagnostic factors relevant to the aim of our study (e.g., internalizing factors predicting the stability of the internalizing class, externalizing class, and so on). The odds ratios indicate the magnitude of the effects of transdiagnostic factors on homotypic or heterotypic continuity of comorbidity classes over time. In terms of homotypic continuity, the internalizing and externalizing factors predicted the stability of the internalizing class, although the magnitude of the effect was greater for internalizing (OR = 7.70; 95% CI: 5.54–10.69) than for externalizing (OR = 3.03; 95% CI: 2.38–3.86). Only the internalizing factor significantly predicted the stability of the externalizing class. Specifically, high internalizing factor scores were associated with increased probabilities of remaining in the externalizing class across time (OR = 1.79; 95% CI: 1.00–3.18) relative to the probabilities of transitioning into the low psychopathology class. In contrast, the effect of the externalizing factor scores on the stability of the externalizing class was non-significant (OR = 1.63; 95% CI: 0.79–3.54; *p* = 0.14).

**TABLE 3:** Log odds coefficients and ratios (OR) with 95% confidence intervals (CI) of the effects of internalizing and externalizing on homotypic and heterotypic continuity.

Transition direction	Covariates							
	INT FS				EXT FS			
	Logit	SE	*z*	OR (95% CI)	Logit	SE	*z*	OR (95% CI)
**Homotypic continuity^a^**								
INT to INT	2.04**	0.17	12.18	7.7 (5.54–10.69)	1.11**	0.12	8.96	3.03 (2.38–3.86)
EXT to EXT	0.58*	0.29	1.97	1.79 (1.00–3.18)	0.49	0.37	1.32	1.63 (0.79–3.54)
**Heterotypic continuity^b^**								
INT to EXT	2.82**	0.33	8.45	16.74 (8.71–32.19)	2.85**	0.27	10.64	17.30 (10.23–29.24)
EXT to INT	2.09**	0.40	5.25	8.07 (3.70–17.60)	0.78	0.53	1.48	2.19 (0.77–6.19)

We conducted a latent transition analysis of three-class common mental disorder comorbidity, including internalizing factor scores, externalizing factor scores, gender, and age as covariates.

^a^Homotypic continuity within internalizing/externalizing class.

^b^Heterotypic continuity across internalizing/externalizing class.

INT, internalizing class; EXT, externalizing class; INT FS, internalizing factor scores; EXT FS, externalizing factor scores.

*p < 0.05; **p < 0.01.

Regarding heterotypic continuity, internalizing and externalizing significantly predicted the transition from the Wave 1 internalizing class to the Wave 2 externalizing class, where the magnitude of the effect was similar, but slightly greater for externalizing (OR = 17.30; 95% CI: 10.23–29.24) than for internalizing (OR = 16.74; 95% CI: 8.71–32.19). However, only the internalizing factor predicted the transition from the Wave 1 externalizing class to the Wave 2 internalizing class. That is, high internalizing factor scores were associated with increased probabilities of transitioning from the externalizing class to the internalizing class (OR = 8.07) relative to the probabilities of transitioning into the low psychopathology class; the effect of the externalizing factor scores on that transition was not significant (OR = 2.19; 95% CI: 0.77–6.19; *p* = 0.19).

## 4. Discussion

The primary aim of our study was to examine the utility of transdiagnostic factors (i.e., internalizing and externalizing) in predicting the stability of and transition between internalizing and externalizing comorbidity classes over time based on transdiagnostic approaches. Our overall results indicated that individuals were classified into different comorbidity classes characterized by either elevated levels of either internalizing or externalizing disorders. Furthermore, the stability of and transitions between these comorbidity classes were predicted by transdiagnostic internalizing and externalizing factors. Specifically, internalizing was a significant predictor of (a) maintaining the internalizing class across waves and (b) transitioning from the externalizing class to the internalizing class over time, which was consistent with our hypotheses. On the other hand, over time, externalizing was a significant predictor of transitioning from the internalizing to the externalizing class. Notably, our results also revealed more dynamic prediction patterns for the continuity of psychopathology symptoms. For example, internalizing contributed to (a) the stability of the externalizing class across waves and (b) the transition from the internalizing class to the externalizing class over time. In contrast, externalizing contributed to the stability of the internalizing class over time. In our opinion, these findings offer a strong and multi-method approach to comprehending the comorbidity and stability of common mental disorders through person-centered modeling approaches (i.e., LCA and LTA), as they supplement earlier findings from variable-centered studies. We discuss the implications of these findings in more detail below.

### 4.1. The predictive validity of transdiagnostic factors

Overall findings demonstrated the validity of transdiagnostic factors in predicting homotypic and heterotypic continuity of psychopathology symptoms over time. Regarding homotypic continuity, elevated internalizing factor levels significantly predicted an increased risk of maintaining internalizing psychopathology over time (OR = 7.70) relative to the transition to the low psychopathology class. This finding indicated that the underlying liability to internalizing psychopathology may cause individuals to (a) experience stable mood and anxiety disorder symptoms and/or (b) shift across diagnostic boundaries within the internalizing domain over time. This finding was consistent with previous findings that anxiety disorders predicted subsequent MDD (4, 72, 73) and vice versa (74, 75), which might be attributed to the stability of the internalizing factor. For instance, studies documented that repetitive negative thinking (e.g., rumination and worry), which is regarded as a transdiagnostic factor that accounts for the overlap in internalizing disorders (76–78), fully mediated the future relationship between depression and anxiety (78). This finding suggests that transdiagnostic internalizing, rather than categorical diagnoses, may be a more robust predictor for the homotypic continuity of internalizing psychopathology.

As for heterotypic continuity, the increase in levels of the dimension one is transitioning to (e.g., the externalizing factor score of an individual who transitioned from the internalizing class at Wave 1 to the externalizing class at Wave 2) strongly predicted heterotypic continuity of comorbidity classes over time. For example, although an individual may have an internalizing disorder(s), it is possible that the individual’s latent externalizing liability subsequently leads to their transition to externalizing psychopathology. This finding aligns with prior studies that revealed the moderating effect of trait impulsivity in the association of social anxiety (S.A.) with AUD (79), indicating that S.A. patients with high vulnerability to externalizing psychopathology are at increased risk of developing AUD. In addition, externalizing mediated the relationship between internalizing and subsequent alcohol use problems (80), indicating that those with internalizing issues are likely to transition to AUD through the effect of externalizing over time. These findings suggest that the vulnerability to externalizing psychopathology may underlie the transition from internalizing symptoms to externalizing problems.

In addition, our finding suggests that an individual’s latent internalizing liability may lead to transitioning from an externalizing disorder(s) to internalizing psychopathology. This conclusion is congruent with a previous finding that rumination, which accounts for the temporal co-occurrence of internalizing disorders, may explain the association of aggressive behaviors with subsequent increases in anxiety symptoms (78). Similarly, Lee and Stone (81) argued that the link between externalizing problems and ensuing internalizing problems might be attributable to individuals’ negative self-concepts (i.e., low self-esteem or negative self-evaluation), which is a transdiagnostic dysfunctional cognition shared among internalizing disorders (82–84). These findings indicate that inter-domain transitions between internalizing and externalizing psychopathology may be attributable to transdiagnostic internalizing and externalizing, given their notable predictive validity for heterotypic continuity of psychopathology symptoms.

Overall, these findings provide supporting evidence for the clinical utility of transdiagnostic factors in predicting how one’s mental disorder symptoms will progress over time. For example, our result suggests that clinicians may inaccurately predict the prognosis of a given patient’s symptoms if merely relying on current diagnoses. Since one’s transdiagnostic factor levels can inform the progress of an individual’s recent diagnosis, clinicians should consider the clinical utility and validity of transdiagnostic factors in predicting a patient’s prognosis, often obscured by diagnostic instability and/or comorbidity issues (12, 57, 85–87).

### 4.2. Dynamics of homotypic and heterotypic continuity of internalizing and externalizing psychopathology over time

Although the above findings align with the developmental progression model that anticipates individuals to move in and out of distinct diagnoses depending on their transdiagnostic vulnerability (60), we also found other dynamic patterns of prediction for homotypic and heterotypic continuity. First, the externalizing factor scores predicted the stability of the internalizing class across waves. This finding suggests that externalizing is a predictor—though relatively smaller in effect than internalizing—that increased the probability of maintaining internalizing psychopathology over time. Such implication is congruent with previous reports that chronic stimulant dependence precipitated affective symptoms in patients with depression (88). In addition, researchers found an association between SUDs and the maintenance of bipolar disorder and mood destabilization in patients with affective disorders (89, 90).

A possible explanation for externalizing contributing to the stability of mood and anxiety symptoms over time is the similar underlying neurobiological mechanisms observed in internalizing and externalizing disorders (90, 91). For instance, a study revealed that stimulants-induced alterations in monoamine systems (e.g., serotonin and dopamine systems) were similar to neurobiological changes found in depression, thereby initiating or exacerbating depressive symptoms (90). Additionally, overt expressions of irritability or aggressiveness rooted in vulnerability to externalizing psychopathology were associated with more severe depressive symptoms, greater impairment, and a more chronic course of illness in patients with depression (92). Consistent with these findings, our result indicates that the externalizing liability factor contributes to the maintenance of mood and anxiety disorders, suggesting the importance of additionally assessing one’s externalizing symptoms when evaluating the prognosis of a patient with internalizing disorders.

Another dynamic pattern found was that the internalizing factor scores, but not the externalizing factor scores, predicted the stability of the externalizing class. This discovery indicates that vulnerability to internalizing psychopathology may contribute to maintaining psychological problems characterized by impulsivity, aggression, and substance use. This result is in line with prior findings that high levels of negative emotionality in adolescence are associated with persistent trajectories of alcohol dependence later in life. Meier et al. (93). Another study also revealed an association between early internalizing symptoms and a fast transition through more pathological stages of alcohol use after the onset of drinking, even after controlling for the effect of externalizing symptoms (94). These findings speak to the need to address individuals’ internalizing symptoms as a potent risk factor for the stability of externalizing disorder symptoms.

Finally, the internalizing factor scores, though relatively smaller in effect than externalizing, predicted a transition from the internalizing to the externalizing class over time. One possible explanation for this phenomenon is the “self-medication hypothesis,” which suggests that individuals with affective disorders may turn to alcohol and drugs as a way to alleviate distress (95). Germane to this hypothesis, a study found a prospective association between the severity of emotional disorders and AUD (96). This finding also supports prior studies that examined the role of rumination as a transdiagnostic internalizing factor underlying the association between internalizing symptoms and externalizing behaviors (78, 96). Specifically, the studies revealed that rumination mediated the relationship between depression and subsequent aggressive behaviors (78). These findings suggest that vulnerability to internalizing psychopathology can confer risk for developing subsequent AUD and impulsive behaviors in individuals with emotional disorders.

Overall, our results suggest that increased levels of a given transdiagnostic dimension (e.g., internalizing) can subsequently initiate or exacerbate symptoms of other dimensions (e.g., externalizing), creating dynamic patterns of continuity of psychopathology symptoms over time. This result is also consistent with the notions of multifinality and equifinality. For example, one’s broad vulnerability to internalizing problems can lead to internalizing and externalizing problems (i.e., multifinality), and one’s future internalizing psychopathology may be attributed to either transdiagnostic internalizing or externalizing (i.e., equifinality). These findings indicate that there may be interindividual variability in how transdiagnostic factors affect the trajectories of mental disorder symptom manifestations over time, resulting in multifinality and equifinality of psychopathology symptoms.

### 4.3. The clinical utility of transdiagnostic factors

Our primary finding that transdiagnostic factors predicted the homotypic and heterotypic continuity of psychopathology underscores the importance of incorporating the transdiagnostic approach into assessment and treatment. Considering the notable predictive validity of transdiagnostic factors, internalizing and externalizing may help predict future disorders’ development based on individuals’ transdiagnostic factor levels. A patient’s illness course may vary depending on their broad vulnerability to experiencing internalizing and externalizing psychopathologies over and beyond diagnoses at a particular moment. For instance, if individuals with MDD have increased vulnerability to internalizing disorders, the MDD symptoms would remain stable or transition to other conditions within the internalizing domain over time. In contrast, individuals with MDD and elevated vulnerability to externalizing psychopathologies would subsequently transition to an externalizing mental disorder.

Moreover, given that internalizing predicted the stability of externalizing psychopathology and vice versa, clinicians can utilize transdiagnostic factors to monitor subliminal symptoms that may contribute to the chronicity of patients’ current diagnoses over time. For example, suppose that there is an MDD patient who has subliminal externalizing symptoms due to an increased latent externalizing factor level. It is possible that the person experiences stable or worsening MDD symptoms as well as other internalizing symptoms over time and/or starts experiencing subsequent externalizing problems given the elevated externalizing level. Therefore, clinicians should consider the overall profile (i.e., elevations of transdiagnostic factor levels) of the patient’s psychopathology symptoms, in addition to individual diagnoses to promote optimal assessment and intervention.

In sum, our study is first to investigate the predictive validity of transdiagnostic internalizing and externalizing in the continuity of common mental disorder symptoms over time, using a nationally representative sample of the United States. Overall, our findings indicate that transdiagnostic factors can inform clinicians of how patients’ psychopathology symptoms will progress over time. Therefore, we expect that utilizing transdiagnostic factors in clinical settings will substantially complement the shortcomings of categorical diagnostic systems. In addition, the transdiagnostic approach can provide clues to the prognosis of patients’ mental disorders, enabling clinicians to assess patients’ symptoms accurately and establish appropriate treatment plans.

The current study has several limitations. First, caution is needed when interpreting the homotypic and heterotypic continuity, as the transition between classes may be under regression effects due to the longitudinal design of our study. Further studies need to take into account such regression effects. Second, only 12 common past-year and lifetime mental disorders were used in our analysis to estimate comorbidity classes and transdiagnostic factors. Particularly, the indicators for the externalizing class and externalizing factor were mainly SUDs. Third, the mental disorder diagnoses included in our study were assessed based on the DSM-IV criteria. Future study needs to further investigate whether analysis including DSM5 diagnoses replicates our findings. Fourth, all the indicators used to estimate transdiagnostic internalizing and externalizing were dichotomous diagnoses. While the results of most studies on mental disorder comorbidity using categorical indicators tend to align with those that use symptom-level analysis (97), it would be important for future studies to replicate our findings using symptom-level data (e.g., the severity of symptoms measured using a Likert-type scale).

## Data availability statement

The original contributions presented in this study are included in the article/Supplementary material, further inquiries can be directed to the corresponding author.

## Ethics statement

The studies involving human participants were reviewed and approved by the United States Census Bureau and the Office of Management and Budget. The patients/participants provided their written informed consent to participate in this study.

## Author contributions

HK developed the study concept and provided critical revisions of the manuscript. BJ performed the data analysis and interpretation under the supervision of HK. BJ drafted the manuscript. Both authors contributed to the study design and approved the final version of the article for submission.
