# Supplementary material for: The validity of transdiagnostic factors in predicting homotypic and heterotypic continuity of psychopathology symptoms over time
Source: Front Psychiatry. 2023 May 19;14:1096572. doi: 10.3389/fpsyt.2023.1096572 (PMC10235495; doi:10.3389/fpsyt.2023.1096572)
Supplement: Supplementary file 1 [file Table_1.DOCX]

**Table S1.** Model fit indices for transdiagnostic internalizing and externalizing factors.

*Note:* Correlated CFA: Confirmatory factor analysis model where we allowed internalizing and externalizing to be correlated. Uncorrelated CFA: confirmatory factor analysis model where we constrained internalizing and externalizing to be uncorrelated. ESEM: exploratory structural equation modeling model where we extracted internalizing and externalizing with an orthogonal rotation, CFI: Comparative Fit Index, TLI: Tucker-Lewis Index, RMSEA: Root Mean Square Error of Approximation.
